# Photonics for Bioapplications: Sensors and Technology

**DOI:** 10.3390/bios15070416

**Published:** 2025-07-01

**Authors:** Nélia Alberto, Maria Fátima Domingues, Nunzio Cennamo, Adriana Borriello

**Affiliations:** 1 Instituto de Telecomunicações, University of Aveiro, Campus Universitário de Santiago, 3810-193 Aveiro, Portugal; 2 Department of Biomedical Engineering and Biotechnology, Khalifa University of Science and Technology, Abu Dhabi P.O. Box 127788, United Arab Emirates; 3 Healthcare Engineering Innovation Group, Khalifa University of Science and Technology, Abu Dhabi P.O. Box 127788, United Arab Emirates; 4 Department of Engineering, University of Campania Luigi Vanvitelli, Via Roma 29, 81031 Aversa, Italy; 5 Department of Precision Medicine, University of Campania “Luigi Vanvitelli”, Via De Crecchio 7, 80138 Naples, Italy

Over the past decade, interest in advancing photonic systems for bioapplications has been steadily growing, and various key factors have driven this trend. One major factor is the rapid progress in photonic technology—effects like miniaturization, increased sensitivity, and improved resolution—which has broadened how these systems can be used.

Another driving force is the rising demand for more precise diagnostics. As the need for non-invasive, real-time, and highly sensitive diagnostic tools increases, photonic systems have become crucial, especially in areas like biosensors and point-of-care devices. Furthermore, new applications have emerged; photonic technologies are now being used in fields such as environmental monitoring, biochemical analysis, biomedical imaging, and personalized medicine, further expanding their reach and significance.

Additionally, the development of new materials and bio-receptors, smart sensing bio-surfaces, innovative sensor designs, and related sensing instrumentation have significantly enhanced the performance of photonics systems. These advancements have made the technology more suitable and adaptable to complex bioapplications. The interdisciplinary nature of this field has also contributed to its development. By merging photonics with biology, chemistry, and medicine, researchers from different scientific backgrounds are finding new opportunities to collaborate and innovate. Overall, this increasing interest in photonic systems reflects their potential to address major challenges in healthcare, environmental science, and biotechnology.

This Special Issue was designed to showcase the latest innovations in photonic sensing and interrogation systems for bioapplications. It addressed recent technological advancements and novel materials to smart bio-surfaces, emerging applications, innovative sensor designs, and cutting-edge instrumentation. Contributions included original research articles, communications, and review papers, aiming to provide a snapshot of the current state of research and the possible future directions for the field. The goal was to create a high-quality collection of papers on the following, or related, key topics:

- Optical fiber biosensing/immunosensors;
- New bio/chemical probes for bioapplications;
- Wearable biomedical sensors;
- Point-of-care devices;
- Molecular diagnosis;
- Biomarker detection;
- Imaging sensors;
- Optical sensors in e-Health architectures;
- Optical chemical sensors;
- Energy-efficient eHealth architecture;
- Big data analysis for eHealth;
- Plasmonic sensors and interrogation systems;
- Molecularly imprinted polymers for sensing;
- Advanced signal processing techniques;
- Photonic integrated circuits (PICs) for bioapplications;
- Low-cost, miniaturized, selective, and multiparameter photonic devices.

The contributions to this Special Issue resulted in a collection of 11 manuscripts that report advances in photonics for bioapplications, emphasizing the relevance of the topic to the research community and showcasing the diverse applications where these innovative technological developments can enhance both performance and impact.

For instance, Yin et al. developed an enzyme-assisted fluorescent biosensor that utilizes circular single-stranded DNA for miRNA detection (Contribution 1). This device reaches a detection threshold as low as 0.36 nM, outperforming many previous miRNA sensors and demonstrating robust detection even in complex samples like serum, making it a valuable tool for early disease diagnosis.

Trypsin is recognized as a potential biomarker in cancers like colorectal, gastric, and pancreatic. A novel near-infrared sensitive dye–peptide conjugate (SQ-3 PC) for selectively detecting trypsin activity via fluorescence ON/OFF sensing is proposed (Contribution 2). The probe exhibits high sensitivity, a linear response up to 30 nM, and a limit of detection and limit of quantification of 1.07 nM and 3.25 nM, respectively. The device demonstrates strong selectivity for trypsin, even in the presence of other enzymes, making it suitable for complex biological applications.

The communication proposed by Xu et al. reports a new THz metasurface sensor for cinnamoylglycine detection, a urinary biomarker of gestational diabetes mellitus (GDM) (Contribution 3). The device, produced from lithium tantalate prism tetramers on quartz, achieves a high Q-factor of 231, enhancing molecular detection. Simulations show a detection limit of 1.23 μg × cm^−2^, demonstrating excellent sensitivity. Given the risks associated with GDM, like preeclampsia and preterm birth, the sensor offers a faster and more accurate alternative to traditional tests, with potential for detecting other biomarkers and monitoring disease progression.

Park et al. developed an *Asterias forbesi*-inspired surface-enhanced Raman spectroscopy (SERS) with numerous hotspots (Contribution 4). The substrate showed strong SERS performance, minimized oxidation damage, and ensured repeatability for uric acid detection. Under laboratory conditions, uric acid was detected at concentrations as low as 1.16 nM, and selectivity was demonstrated against various metabolites. In a simulated real-world scenario, the sensor successfully detected a wide range of uric acid concentrations, encompassing both deficiency and excess levels, in serum and urine samples.

In the study presented by Jiao et al., the polarimetric aberrations caused by three different illumination schemes in backscattering Mueller Matrix imaging were analyzed (Contribution 5). The optimized schemes established key criteria for selecting spatial illumination patterns in non-collinear backscattering Mueller Matrix measurements, offering valuable insights for advancing quantitative tissue polarimetric imaging and biosensing.

In (Contribution 6), the viscoelastic properties of AuNP-assisted radiofrequency-heated and ablated tissues were analyzed, correlating them with temperature profiles using confocal Brillouin micro-spectroscopy and Mg-silicate-NP-doped temperature-sensing fibers. The results reveal that changes in the Brillouin peak were linked to thermal doses and protein denaturation. The hybrid Brillouin–optical backscattering reflectometry technique shows potential for monitoring tissue deformations during thermal therapies, enabling the real-time tracking of viscoelastic and thermal properties in metal-nanoparticle-embedded tissues for hyperthermal and theranostic applications.

A red-light-driven photoelectrochemical (PEC) sensor based on a g-C_3_N_4_/CuS/TiO_2_ ternary heterojunction for the sensitive detection of the synergistic antioxidant effects of sesamol and other antioxidants is reported in (Contribution 7). Sesamol is a natural antioxidant that reduces free radicals and supports brain function, improving memory and cognition in Alzheimer’s patients. By scavenging holes in the valence band, the antioxidants suppress charge carrier recombination, boosting the PEC photocurrent. The sensor exhibited high sensitivity, selectivity, and stability, when applied to detect sesamol in soybean and peanut oils. It holds potential for applications in nutrition analysis, food fraud detection, and medical research, contributing to improved consumer health and product quality.

Zhou et al. developed titanium metal–organic framework (Ti-MOF) microflowers for the detection and removal of Al(III) ions in water (Contribution 8). Ti-MOF offers high water stability, porosity, and strong luminescence, with a detection range of 0.4–15 µM and a limit of 75 nM, well below the World Health Organization’s aluminum safety limit. It also allows selective Al(III) detection by shielding interference from Fe(III) using ascorbic acid, making it an effective sensor and adsorbent for water purification.

A nano-slit array sensor based on temperature variation is proposed for the highly sensitive and specific detection of molecular fingerprints (Contribution 9). As the temperature changes, the dielectric properties of the temperature-sensitive semiconductor material InSb dynamically adjust, causing shifts in the transmission resonance angle and producing transmission envelope curves over a broad frequency range. Using this temperature scanning approach, the characteristic fingerprint spectra of hexogen, a high-impact military explosive, were successfully identified, allowing both qualitative and quantitative detection with a sensitivity limit of 1.61 μg/cm^2^.

A distributed optical fiber sensing network was proposed in (Contribution 10) for the two-dimensional in situ thermal mapping of advanced methods for radiofrequency thermal ablation. Radiofrequency ablation was conducted on a bovine phantom using a standard approach, while methods enhanced with agarose and gold nanoparticles were carried out to improve the ablation efficiency. Results showed that agarose-mediated thermal ablation treated the largest area, but with low repeatability, while AuNP-mediated ablation at a 4 mg/mL density offered the best balance between ablation efficacy and repeatability.

The Special Issue concludes with a review paper dedicated to cost-effective optical fiber-based biosensing platforms, exploring various geometries, interrogation techniques, and encapsulation methods (Contribution 11). The potential of these cost-effective optical fiber sensing techniques is highlighted through numerous successful applications, in three key areas: cancer, cardiovascular biomarker detection, and environmental monitoring. Some weaknesses of these systems are also highlighted, emphasizing the need to improve aspects related to repeatability and reproducibility, as well as the development of techniques for sensor reuse.

In conclusion, this Special Issue presents a selection of manuscripts that highlight recent advances in the field of photonics, with a particular focus on bioapplications. The works gathered here reflect the diversity and breadth that characterize both the research domain itself and the applications. Although they represent only a sample of the extensive ongoing developments, the published articles provide a meaningful overview of the current trends and pave the way for future opportunities for research and innovation in the field of photonic sensors, emphasizing their potential contributions to biomedicine and related areas.

We sincerely thank the authors for their valuable contributions to this Special Issue, which brings together original research articles that provide a comprehensive and current overview of a diverse array of topics, all unified by a shared focus on “Photonics for bioapplications: Sensors and Technology”. Additionally, we express our gratitude to the academic editors and reviewers for their dedicated efforts in ensuring the scientific rigor and quality of the published manuscripts. Their efforts have significantly enhanced the overall impact and relevance of this Special Issue.

